# P-980. Development and Implementation of Antimicrobial Stewardship in India: A Meta-synthesis of Pharmacists Perspective

**DOI:** 10.1093/ofid/ofaf695.1179

**Published:** 2026-01-11

**Authors:** Muhammed Rashid, Racha Amarthya Sree, Sathyanarayana Reddy Bygari, Vrinda Nampoothiri, V H Mohammed, Mohamed Hisham, Mohammed Faisal, Payal K Patel

**Affiliations:** Federation of Clinical Pharmacists in India, Bangalore, Karnataka, India; Federation of Clinical Pharmacists in India, Bangalore, Karnataka, India; Federation of Clinical Pharmacists in India, Bangalore, Karnataka, India; Amrita Hospital, Kochi, Ernakulam, Kerala, India; Aster MIMS, Calicut, Kerala, India; Federation of Clinical Pharmacists in India, Bangalore, Karnataka, India; Emirates Health Services, Sharjah, Sharjah, United Arab Emirates; Intermountain Health, Murray, UT, USA, Murray, Utah

## Abstract

**Background:**

There are still barriers in developing and implementing antimicrobial stewardship (AMS) in India and a clear understanding is still lacking. This meta-synthesis aimed to understand pharmacists' experiences regarding the facilitators and barriers of AMS in India.

**Methods:**

PubMed, Scopus, Embase and Cochrane Library were searched from inception to February 2025. Key responsibilities, key challenges and barriers in developing and implementing the AMS in Indian settings were captured fromEnglish language qualitative studies. Only the studies including the participants from India were considered. A preliminary theme followed by the sub-themes were developed from each study and narrative synthesis was performed to synthesize the data.

**Results:**

A total of 5 studies out of 98 non-duplicate records published between 2019 to 2024 were eligible. All the studies included semi-structured interviews with or without focus group discussions with the majority of participants from private settings. There was a country-level heterogeneity in AMS. Pharm.D or MPharm graduates with an informal learning with physicians were mostly practicing AMS in India. Current pharmacy curricula do not provide adequate training in AMS. Key facilitators of AMS at the hospital included a dedicated committee overseeing appropriate inpatient antibiotic use, a prompt microbiology laboratory, a high level of AMS understanding among staff, established guidelines for empiric prescribing and an easily accessible antibiogram. The barriers include the limited access to clinical pharmacists, lack of formal training, physician immunity to change regarding stewardship policies, professional boundaries, infrequent antibiotic de-escalation, high workload and lack of incentives, an incomplete electronic medical record, inadequate AMS physical visibility and high antibiotic use in the community.

**Conclusion:**

A contextually developed, standardized and accessible AMS training programme along with pharmacy curricula modification to include AMS, may facilitate AMS in India. State level support for ASP with additional funding was perceived very essential. The learnings from this study will help the policymakers to improve the AMS development, implementation and practice in India.

**Disclosures:**

All Authors: No reported disclosures

